# Proteomic profiling reveals ACTN1‐mediated cytoskeletal remodelling and hyperplasia of cerebrovascular smooth muscle cells in moyamoya disease

**DOI:** 10.1002/ctm2.70730

**Published:** 2026-07-06

**Authors:** Yaoren Chang, Zhenyu Zhou, Junze Zhang, Yuanli Zhao, Shihao He, Xun Ye

**Affiliations:** ^1^ Department of Neurosurgery Beijing Neurosurgical Institute, Capital Medical University Beijing China; ^2^ Department of Neurosurgery Beijing Tiantan Hospital, Capital Medical University Beijing China; ^3^ Department of Neurosurgery, Peking Union Medical College Hospital Peking Union Medical College and Chinese Academy of Medical Sciences Beijing China; ^4^ Department of Pathology Johns Hopkins School of Medicine Baltimore Maryland USA

Dear Editor,

Through serum proteomic profiling in moyamoya disease (MMD), we identify α‐actinin‐1 (ACTN1) as a candidate protein upregulated in both haemorrhagic and ischaemic MMD. Using overexpression, pharmacological inhibition and knockdown in cerebral vascular smooth muscle cells (VSMCs), including patient‐derived cells, we find that ACTN1 is associated with enhanced proliferation, migration and cytoskeletal reorganization through the RhoA/ROCK and MEK/ERK pathways, processes that may contribute to the intimal thickening that characterizes MMD.

MMD is a rare, progressive cerebrovascular disease characterized by stenosis or occlusion of the terminal internal carotid arteries and their branches.^1^ Its clinical heterogeneity suggests that genetic variation alone does not fully explain its pathogenesis, and proteomic approaches may help clarify the mechanisms. In an early study, Koh et al.^2^ used two‐dimensional electrophoresis to compare the serum proteomes of patients with MMD and controls and reported abnormal complement C1 inhibitor, although the few proteins resolved limited mechanistic insight. Subsequent work has repeatedly implicated cytoskeletal proteins: Wang et al.^3^ found downregulation of cofilin and the ARP2/3 complex in serum exosomes from haemorrhagic MMD, linked to endothelial mitochondrial dysfunction, and He et al.^4^ reported that upregulated cytoskeletal proteins promote pathological angiogenesis in MMD. A circulating protein, serum amyloid A2, has also been reported to drive VSMC phenotypic switching in MMD, indicating that serum proteins can actively shape the vascular phenotype.^5^ Here, data‐independent acquisition (DIA) serum proteomics quantified 1541 proteins and, with validation across both MMD subtypes, identified ACTN1 as a candidate link between altered cytoskeletal dynamics and VSMC remodelling through the RhoA/ROCK and MEK/ERK pathways (Figure 1).

**FIGURE 1:**
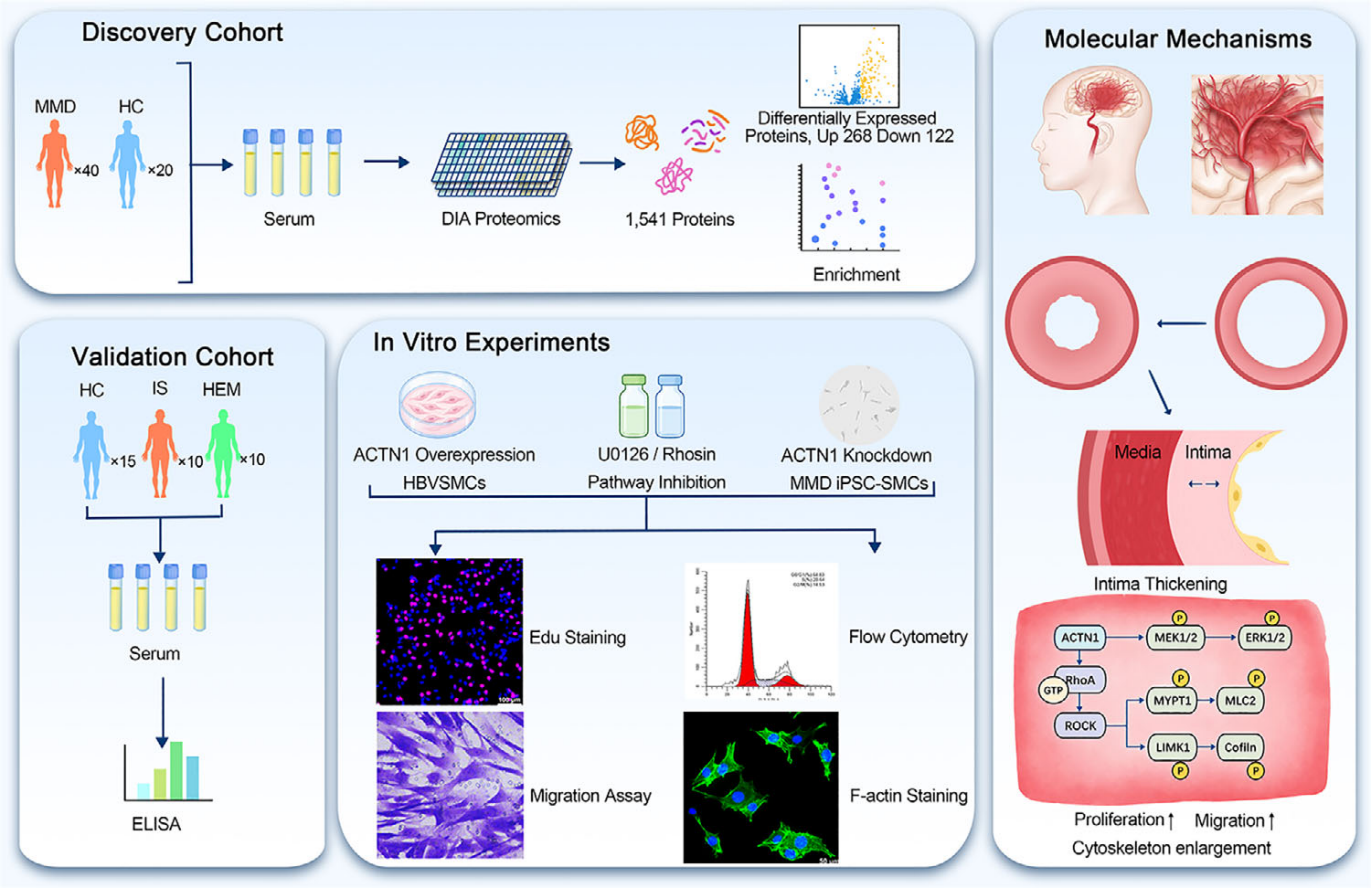
Mechanistic illustration of ACTN1‐associated cytoskeletal remodelling in moyamoya disease.

Among the 1541 quantified proteinsAmong the 1541 quantified proteins in Supplementary Dataset 1, 390 were differentially expressed between patients with MMD and controls (268 upregulated and 122 downregulated), including ACTN1 (Figure 2A). Functional enrichment of these proteins pointed to coagulation‐related processes, extracellular and vesicular compartments, and functional terms related to protease regulation, while Kyoto Encyclopedia of Genes and Genomes (KEGG) analysis highlighted focal adhesion, ECM‐receptor interaction and regulation of actin cytoskeleton (Figure 2B; Figure S1). As an actin‐crosslinking protein implicated in cell adhesion and migration, ACTN1 was selected for further study.^6^ In an independent cohort, enzyme‐linked immunosorbent assay (ELISA) showed higher serum ACTN1 in both haemorrhagic and ischaemic MMD than in controls (Figure 2C). To test whether ACTN1 itself alters VSMC behaviour, we overexpressed it in human brain VSMCs (HBVSMCs) in the absence of MMD serum. Relative to vector controls, ACTN1 overexpression promoted the proliferation, migration and cytoskeletal reorganization of cerebral VSMCs (Figure 2D–K). Elevated ACTN1 alone was sufficient to increase VSMC proliferation and migration, changes also seen during vascular remodelling.^7^

**FIGURE 2:**
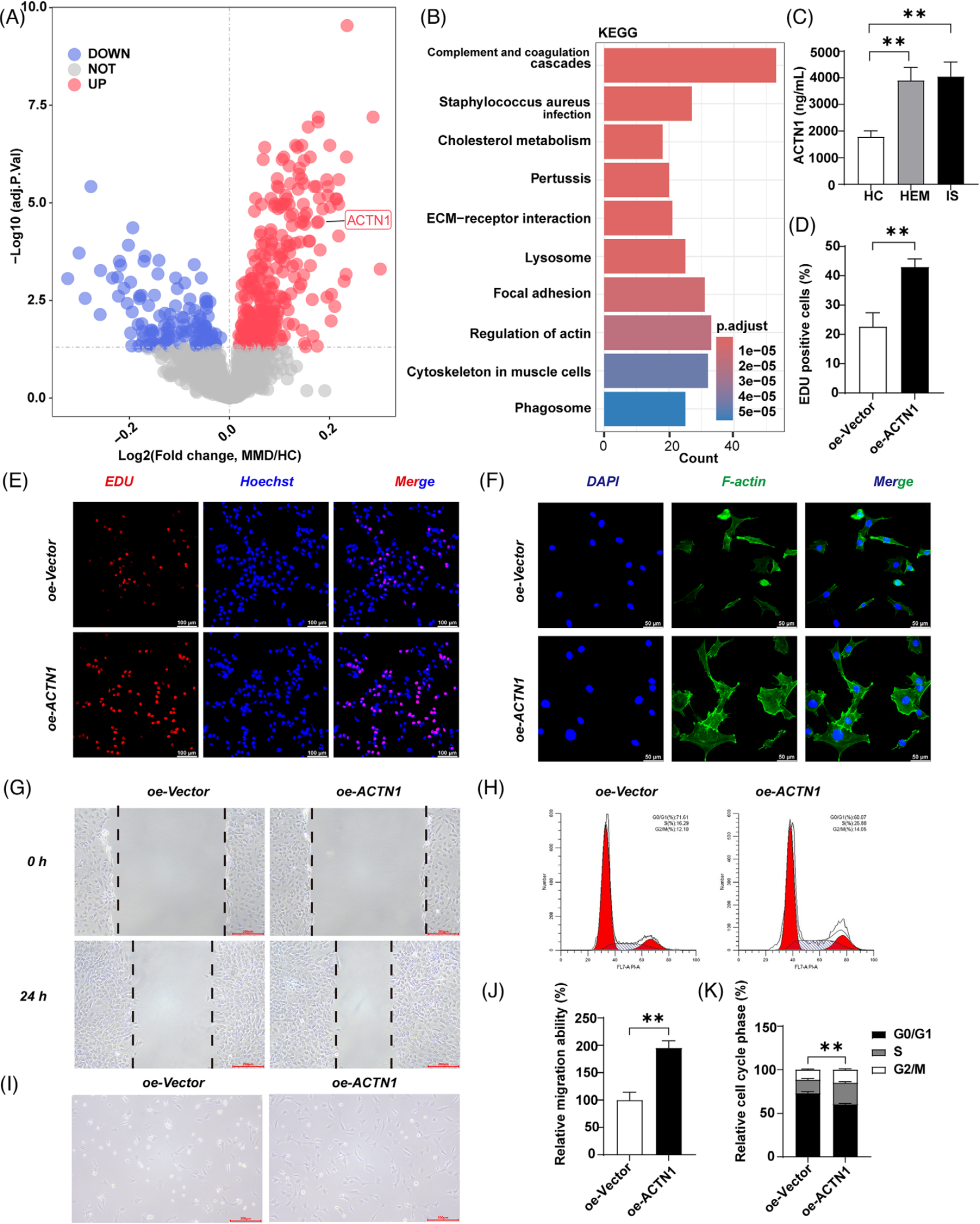
Serum ACTN1 is elevated in HEM and IS and promotes a proliferative and migratory phenotype in HBVSMCs. (A) Volcano plot of serum proteins in patients with MMD versus HC; *x*‐axis, log2 fold change (MMD/HC); ACTN1 is indicated (red, upregulated; blue, downregulated; grey, not significant). (B) KEGG pathway enrichment of the differentially expressed proteins, coloured by adjusted *p* value. (C) Serum ACTN1 measured by ELISA in HC (*n* = 15), HEM (*n* = 10) and IS (*n* = 10). (D) Bar chart showing the proportion of proliferative cells from the EdU assay in oe‐ACTN1 versus oe‐Vector HBVSMCs. (E) EdU (red) and Hoechst (blue) staining of oe‐Vector and oe‐ACTN1 HBVSMCs; scale bar = 100 µm. (F) F‐actin (green) and DAPI (blue) staining; scale bar = 50 µm. (G) Cell scratch assay at 0 and 24 h; scale bar = 200 µm. (H) Flow cytometric cell cycle analysis. (I) Representative bright‐field images showing morphological changes in oe‐Vector and oe‐ACTN1 HBVSMCs; scale bar = 200 µm. (J) Bar chart showing the relative migration of HBVSMCs from the cell scratch assay in (G). (K) Bar chart showing the cell cycle distribution (G0/G1, S, G2/M) from the flow cytometry in (H). Data are mean ± SD. For ELISA in panel C, *n* = 15 for HC, *n* = 10 for HEM and *n* = 10 for IS; for the in vitro experiments in panels D–K, data are from three independent experiments. ^**^ *p *< .01.

To characterize the signalling downstream of ACTN1, we examined the RhoA/ROCK and MEK/ERK pathways. In HBVSMCs, ACTN1 overexpression increased RhoA activation and ROCK expression, together with phosphorylation of their downstream effectors, and increased phosphorylation of MEK1/2 and ERK1/2 (Figure S2). To test whether these pathways mediated the ACTN1‐driven phenotype, ACTN1‐overexpressing cells were treated with the RhoA inhibitor Rhosin or the MEK inhibitor U0126. Both inhibitors attenuated the proliferation, migration and cytoskeletal reorganization induced by ACTN1 (Figure 3). At the molecular level, Rhosin reduced RhoA/ROCK signalling and U0126 reduced MEK/ERK signalling, each with limited effect on the other pathway (Figures S3 and S4). This indicates that both RhoA/ROCK and MEK/ERK are involved in the effects of ACTN1, pathways already known to regulate VSMC proliferation and migration.^8^, ^9^, ^10^

**FIGURE 3:**
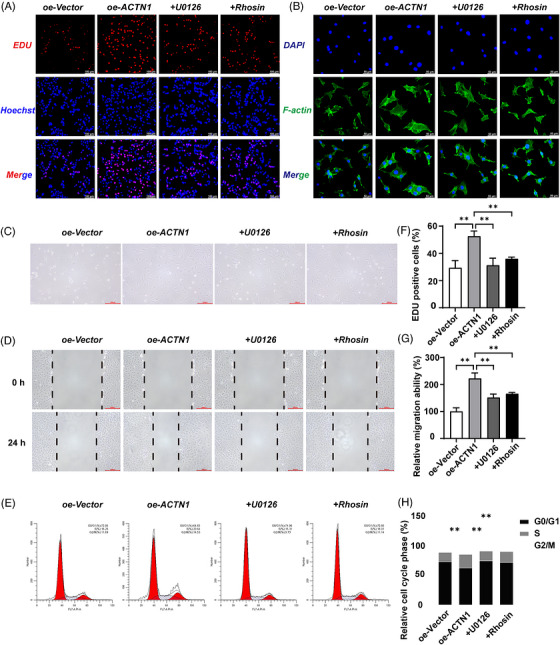
Inhibition of RhoA or MEK attenuates the ACTN1‐associated phenotype in HBVSMCs. +U0126 and +Rhosin denote oe‐ACTN1 HBVSMCs treated with the MEK inhibitor U0126 or the RhoA inhibitor Rhosin, respectively; oe‐Vector and oe‐ACTN1 HBVSMCs were used as controls. (A). EdU (red) and Hoechst (blue) staining; scale bar = 100 µm. (B) F‐actin (green) and DAPI (blue) staining; scale bar = 50 µm. (C) Bright‐field images; scale bar = 200 µm. (D) Cell scratch assay at 0 and 24 h; scale bar = 200 µm. (E) Flow cytometric cell cycle analysis. (F) Bar chart showing the proportion of proliferative cells from the EdU assay. (G) Bar chart showing the relative migration from the cell scratch assay in (D). (H) Bar chart showing the cell cycle distribution (G0/G1, S, G2/M) from the flow cytometry in (E). Data are mean ± SD from three independent experiments. ^**^ *p* < .01.

To study ACTN1 in a patient‐relevant context, we used induced pluripotent stem cell‐derived smooth muscle cells (iPSC‐SMCs) from patients with MMD (MMD‐iPSC‐SMCs) and from healthy controls (HC‐iPSC‐SMCs). Relative to HC‐iPSC‐SMCs, MMD‐iPSC‐SMCs showed greater proliferation, migration and cytoskeletal reorganization, together with higher RhoA/ROCK and MEK/ERK signalling (Figure 4 ; Figure S5). ShRNA‐mediated knockdown of ACTN1 in MMD‐iPSC‐SMCs, but not a non‐targeting control, attenuated these phenotypes and reduced signalling through both pathways (Figure 4; Figure S5). In these patient‐derived cells, lowering ACTN1 reduced both the proliferative and migratory phenotype and the activity of the two pathways. These loss‐of‐function data support a role for ACTN1 in the proliferative and migratory phenotype of patient‐derived MMD‐iPSC‐SMCs and in activation of the RhoA/ROCK and MEK/ERK pathways, consistent with the overexpression and pharmacological inhibition findings.

**FIGURE 4:**
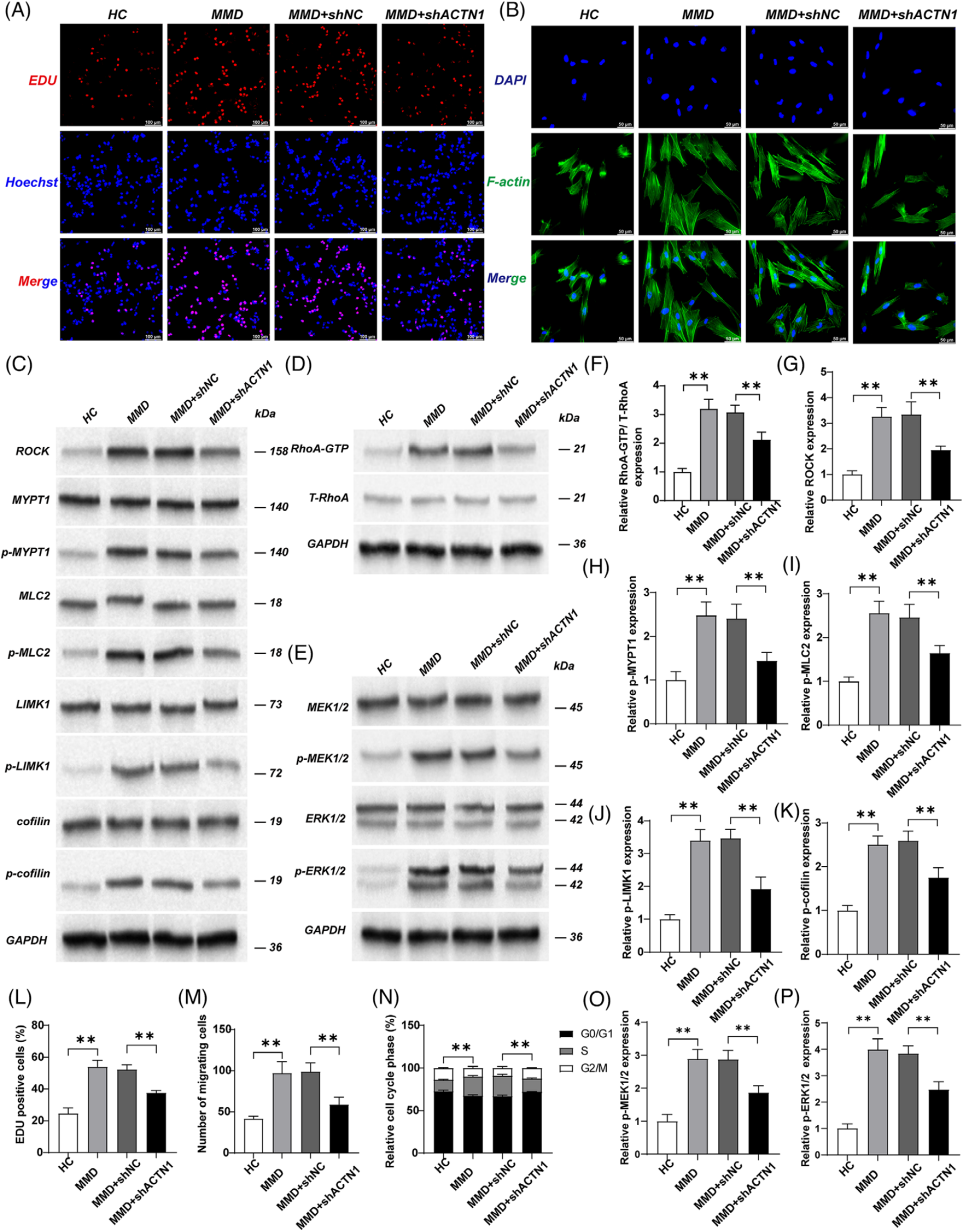
ACTN1 knockdown attenuates the proliferative, migratory phenotype and pathway activation in patient‐derived MMD‐iPSC‐SMCs. HC and MMD denote HC‐iPSC‐SMCs and MMD‐iPSC‐SMCs, respectively. MMD+shNC denotes MMD‐iPSC‐SMCs transfected with a non‐targeting shRNA, and MMD+shACTN1 denotes MMD‐iPSC‐SMCs transfected with shRNA against ACTN1. (A) EdU (red) and Hoechst (blue) staining; scale bar = 100 µm. (B) F‐actin (green) and DAPI (blue) staining; scale bar = 50 µm. (C) Western blots of ROCK, MYPT1, p‐MYPT1, MLC2, p‐MLC2, LIMK1, p‐LIMK1, cofilin, p‐cofilin and GAPDH. (D) Western blots of RhoA‐GTP, total RhoA (T‐RhoA) and GAPDH. (E) Western blots of MEK1/2, p‐MEK1/2, ERK1/2, p‐ERK1/2 and GAPDH. (F–K) Bar charts showing the relative protein expression of (F). RhoA‐GTP/T‐RhoA, (G) ROCK, (H) p‐MYPT1, (I) p‐MLC2, (J) p‐LIMK1 and (K) p‐cofilin. (L) Bar chart showing the proportion of EdU‐positive cells. (M) Bar chart showing the number of migrated cells from the transwell assay. (N) Bar chart showing the cell cycle distribution (G0/G1, S and G2/M). (O and P) Bar charts showing the relative protein expression of (O) p‐MEK1/2 and (P) p‐ERK1/2. Data are mean ± SD from three independent experiments. ^**^ *p* < .01.

These findings identify ACTN1 as a candidate serum protein elevated in both haemorrhagic and ischaemic MMD and support a role for ACTN1 in the proliferation, migration and cytoskeletal reorganization of cerebral VSMCs through the RhoA/ROCK and MEK/ERK pathways. This is supported by three sets of experiments: ACTN1 overexpression was sufficient to induce the phenotype in HBVSMCs, inhibition of RhoA or MEK reversed it, and ACTN1 knockdown in patient‐derived MMD‐iPSC‐SMCs reduced it. These changes may contribute to the intimal thickening that characterizes MMD and nominate ACTN1 as a candidate circulating marker and a molecular target for further evaluation. Several limitations should be noted. The validation cohort was modest, and the serum elevation at the discovery stage was small, so both its magnitude and its diagnostic value require confirmation in larger, independent and clinically stratified cohorts. The cellular models, although including patient‐derived cells, do not reproduce the multicellular architecture and hemodynamic environment of cerebral arteries, and ACTN1 was not examined in vessel tissue. Together, these findings support ACTN1 as a candidate molecule linked to vascular remodelling in MMD, while its potential diagnostic or therapeutic relevance requires further validation in larger patient cohorts, and in vivo models.

In the discovery stage, serum from patients with moyamoya disease (MMD) and healthy controls (HC) was analysed by data‐independent acquisition (DIA) proteomics, and serum α‐actinin‐1 (ACTN1) was validated by enzyme‐linked immunosorbent assay (ELISA) in HC, haemorrhagic MMD (HEM) and ischaemic MMD (IS). ACTN1 was then studied in vitro by overexpression in human brain vascular smooth muscle cells (HBVSMCs), by pharmacological inhibition with the RhoA inhibitor Rhosin or the MEK inhibitor U0126, and by shRNA knockdown in induced pluripotent stem cell (iPSC)‐derived smooth muscle cells from patients with MMD (MMD‐iPSC‐SMCs), using 5‐ethynyl‐2′‐deoxyuridine (EdU), cell scratch assay, flow cytometry and F‐actin assays. Collectively, these experiments suggest that ACTN1 is associated with VSMC proliferation, migration and cytoskeletal reorganization through the RhoA/ROCK and MEK/ERK pathways, processes that may contribute to intimal thickening in MMD.

## AUTHOR CONTRIBUTIONS

Shihao He and Xun Ye conceived and designed the experiments. Yaoren Chang, Junze Zhang, Shihao He and Zhenyu Zhou performed experiments. Yuanli Zhao contributed reagents, materials and analytical tools. All the authors participated in the writing of the manuscript.

## FUNDING INFORMATION

This research was funded by the National Natural Science Foundation of China (grant no.: 82471328), which covered expenses related to testing and processing, data collection, analysis and interpretation of the experimental data.

## CONFLICT OF INTEREST STATEMENT

The authors declare no conflicts of interest.

## ETHICAL APPROVAL

This study was approved by the Institutional Ethics Committee of Beijing Tiantan Hospital (KYSQ2024‐248‐01). All participants signed informed consent forms.

## DISCLOSURES

None.

## Supporting information



Supporting Information: ctm270730‐sup‐0001‐SuppMatdataset.xls

Supporting Information: ctm270730‐sup‐0002‐SuppMat.pdf

## Data Availability

The processed proteomics data generated in this study, including the differential protein tables from the DIA proteomic analysis, are provided as Supplementary Dataset. Any other information presented in the current study are available from the corresponding author on reasonable request.

## References

[1] Scott RM . Moyamoya Disease and Moyamoya Syndrome. n engl j med. 2009;360(12):1226‐1237. 19297575 10.1056/NEJMra0804622

[2] Koh E‐J , Kim H‐N , Ma T‐Z , et al. Comparative analysis of serum proteomes of moyamoya disease and normal controls. J Korean Neurosurg Soc. 2010;48:8‐13. doi:10.3340/jkns.2010.48.1.8 20717506 10.3340/jkns.2010.48.1.8 PMC2916155

[3] Wang X , Han C , Jia Y , et al. Proteomic profiling of exosomes from hemorrhagic moyamoya disease and dysfunction of mitochondria in endothelial cells. Stroke. 2021;52:3351‐3361. doi:10.1161/STROKEAHA.120.032297 34334053 10.1161/STROKEAHA.120.032297

[4] He S , Zhang J , Liu Z , et al. Upregulated Cytoskeletal Proteins Promote Pathological Angiogenesis in Moyamoya Disease. Stroke. 2023;54:3153‐3164. doi:10.1161/STROKEAHA.123.044476 37886851 10.1161/STROKEAHA.123.044476

[5] He S , Zhang J , Zhou Z , et al. Serum amyloid A2–induced phenotypic switching of vascular smooth muscle cells in moyamoya disease. Journal of Neurosurgery. 2025:1‐13. doi:10.3171/2024.11.JNS241804 10.3171/2024.11.JNS241804 40215623

[6] Sjöblom B , Salmazo A , Djinović‐Carugo K . Alpha‐actinin structure and regulation. Cell mol life sci: CMLS. 2008;65:2688‐2701. doi:10.1007/s00018‐008‐8080‐8 18488141 10.1007/s00018-008-8080-8 PMC11131806

[7] Owens GK , Kumar MS , Wamhoff BR . Molecular regulation of vascular smooth muscle cell differentiation in development and disease. Physiol Rev. 2004;84:767‐801. doi:10.1152/physrev.00041.2003 15269336 10.1152/physrev.00041.2003

[8] Shimokawa H , Sunamura S , Satoh K . RhoA/rho‐kinase in the cardiovascular system. Circulation Research. 2016;118:352‐366. doi:10.1161/CIRCRESAHA.115.306532 26838319 10.1161/CIRCRESAHA.115.306532

[9] Kingsley K , Huff JL , Rust WL , et al. ERK1/2 mediates PDGF‐BB stimulated vascular smooth muscle cell proliferation and migration on laminin‐5. Biochem Biophys Res Commun. 2002;293:1001‐1006. doi:10.1016/S0006‐291X(02)00331‐5 10.1016/S0006-291X(02)00331-5 12051759

[10] Zhan Y , Kim S , Izumi Y , et al. Role of JNK, p38, and ERK in platelet‐derived growth factor‐induced vascular proliferation, migration, and gene expression. Arterioscler, Thromb, Vasc Biol. 2003;23:795‐801. doi:10.1161/01.ATV.0000066132.32063.F2 12637337 10.1161/01.ATV.0000066132.32063.F2

