# Supplementary material for: Proteomic profiling reveals ACTN1‐mediated cytoskeletal remodelling and hyperplasia of cerebrovascular smooth muscle cells in moyamoya disease
Source: Clin Transl Med. 2026 Jul 6;16(7):e70730. doi: 10.1002/ctm2.70730 (PMC13338571; doi:10.1002/ctm2.70730)
Supplement: Supplementary file 2 — Supporting Information: ctm270730‐sup‐0002‐SuppMat.pdf [file CTM2-16-e70730-s002.pdf]

Supplementary materials

Methods

Study participants and serum sample collection

A total of 40 patients diagnosed with moyamoya disease (MMD) and 20 age- and sex-matched healthy controls (HCs) were included in the discovery cohort. Patients with MMD included 20 patients with hemorrhagic MMD (HEM) and 20 patients with ischemic MMD (IS). All participants were recruited from Beijing Tiantan Hospital, Capital Medical University. The diagnosis of MMD was based on digital subtraction angiography (DSA) or magnetic resonance angiography (MRA), according to established diagnostic guidelines. This study was approved by the Ethics Committee of Beijing Tiantan Hospital, Capital Medical University. Written informed consent was obtained from all participants, or from the parents or legal guardians of participants younger than 18 years, before sample collection.

Peripheral venous blood samples were collected from all participants before revascularization surgery using standard serum collection tubes. After collection, blood samples were kept upright and allowed to clot at room temperature for 30–60 min. Serum was separated by centrifugation at 1,500g for 10 min at room temperature. The upper serum fraction was carefully aspirated without disturbing the clot or cellular interface and transferred into sterile, low-protein-binding microcentrifuge tubes.

All serum samples were visually inspected before aliquoting. Samples with gross hemolysis, obvious turbidity, clot disruption, or visible cellular carryover were excluded from downstream proteomic and ELISA analyses. Serum aliquots were immediately stored at

−80°C until analysis, and repeated freeze–thaw cycles were avoided. The same sample-processing workflow was applied to all MMD and healthy control samples.

Proteomic profiling and bioinformatic analysis by data-independent acquisition

Data-independent acquisition (DIA) quantitative proteomics was used to identify differences in serum protein expression among the HEM, IS, and HC groups. Serum protein samples were denatured by adding protein lysis buffer to serum samples at a sample-to-lysis buffer ratio of 1:9 (w/w). Dithiothreitol was added, and samples were incubated at 37°C for 30 min. After cooling to room temperature, 0.55 M iodoacetamide was added, and samples were incubated for 30 min at room temperature in the dark. Proteins were enriched using solid-phase extraction C18 columns. After extraction and purification, protein samples were redissolved in 25 μL of 50 mM NH4HCO3, vortexed for 1 min, and centrifuged in a microcentrifuge for 1 min. Protein concentrations were determined using Bradford reagent, and protein purity was assessed by sodium dodecyl sulfate-polyacrylamide gel electrophoresis (SDS-PAGE).

Protein digestion was performed by adding trypsin to the protein solution at an enzyme-to-protein ratio of 1:20 (w/w), followed by incubation for 14–16 h at 37°C. For high-pH reversed-phase separation, equal amounts of peptides from each sample were pooled. A total of 20 μg of the peptide mixture was diluted with 2 mL of mobile phase A (5% acetonitrile, pH 9.8) for injection. High-pH reversed-phase separation was performed using a Shimadzu LC-20AB high-performance liquid chromatography system (Shimadzu, Kyoto,

Japan) coupled with a Gemini high-pH C18 column. The resulting fractions were combined into 10 fractions and lyophilized.

Peptides separated by liquid chromatography were ionized using a nano-electrospray ionization source (Advion Triversa Nanomate) and analyzed using an Orbitrap Exploris 480 tandem mass spectrometer (Thermo Fisher Scientific, San Jose, CA, USA) for data-dependent acquisition library construction and DIA mass spectrometry. Data-dependent acquisition data were searched using the Andromeda search engine in MaxQuant for spectral library construction. Large-scale DIA data were processed using the mProphet algorithm for analytical quality control and quantitative analysis. Gene Ontology (GO), Kyoto Encyclopedia of Genes and Genomes (KEGG), and pathway functional annotation analyses were performed. Protein–protein interaction analysis was performed using STRING software (version 3.2.1; https://string-db.org/). Bioinformatic analyses were performed using R software (version 3.4; R Foundation for Statistical Computing). DIA proteomic analysis was performed by Beijing Genomics Institute (BGI, Shenzhen, China).

Validation of serum ACTN1 by enzyme-linked immunosorbent assay

Serum α-actinin-1 (ACTN1) levels were measured by enzyme-linked immunosorbent assay (ELISA) using a Human ACTN1 ELISA kit (ABIN6953408; Antibodies Online, PA, USA) according to the manufacturer's instructions. Serum samples from the HC, HEM, and IS groups were used for validation. ACTN1 standards were prepared at concentrations of 20, 10, 5, 2.5, 1.25, 0.625, 0.312, and 0 ng/mL.

Briefly, 100 μL of standard or serum sample was added to each well of the pre-coated microplate and incubated at 37°C for 1 h. After discarding the liquid, 100 μL of assay reagent A was added to each well and incubated at 37°C for 1 h. The plate was washed three times with 350 μL washing buffer, with 1 min soaking for each wash. Subsequently, 100 μL of assay reagent B was added to each well and incubated at 37°C for 30 min. After five additional washes, 90 μL of substrate solution was added to each well and incubated at 37°C for 15 min in the dark. The reaction was terminated by adding 50 μL of stop solution. Optical density was measured immediately at 450 nm using a full-wavelength microplate reader (HBS-SCANX; Nanjing Detie Biotechnology, Nanjing, China).

Cell culture

Human brain vascular smooth muscle cells (HBVSMCs; 1106) were purchased from ScienCell Research Laboratories. HBVSMCs were cultured in smooth muscle cell medium (SMCM; 1101; ScienCell Research Laboratories) supplemented with 5% fetal bovine serum (FBS; SH30070.03; Hyclone, Logan, UT, USA), 1% smooth muscle cell growth supplement (SMCGS; 1152; ScienCell Research Laboratories), and 1% penicillin–streptomycin solution (15140148; Thermo Fisher Scientific, Pittsburgh, PA, USA). Cells were maintained in a humidified incubator (Forma 3111; Thermo Electron, Waltham, MA, USA) at 37°C with 5% CO2.

Cells were passaged when they reached approximately 90% confluence. Briefly, the culture medium was removed, and cells were washed twice with phosphate-buffered saline (PBS). Cells were then digested using 0.25% trypsin-0.02% EDTA (15090046; Gibco, Grand Island,

NY, USA). When the cells became rounded under microscopic observation, digestion was terminated by adding complete medium. The cell suspension was collected by gentle pipetting and centrifuged at 800 rpm for 5 min at 4°C before reseeding.

Healthy control induced pluripotent stem cell-derived smooth muscle cells (HC-iPSC-SMCs) and moyamoya disease induced pluripotent stem cell-derived smooth muscle cells (MMD-iPSC-SMCs) were used as donor-derived smooth muscle cell models.
HC-iPSC-SMCs and MMD-iPSC-SMCs were maintained in smooth muscle cell-specific culture medium under standard culture conditions. These cells were passaged at approximately 90% confluence using the same trypsinization and reseeding procedure.

ACTN1 overexpression in HBVSMCs

ACTN1 overexpression in HBVSMCs was performed using a lentiviral vector system. The coding sequence of ACTN1 was amplified using gene-specific primers and cloned into the pLVX-Puro vector (VT1465; YouBio, Hunan, China). The upstream primer was 5′-GGAATTCCATGGACCATTATGATTCTCAGC-3′, and the downstream primer was 5′-CTCTAGAATTAGAGGTCACTCTCGCCGTA-3′. EcoRI and XbaI restriction sites were introduced at the ends of the primers. Restriction digestion was performed using EcoRI and XbaI, and ligation was performed using T4 DNA ligase (Takara, Japan).

The restriction digestion reaction was performed in a 50-μL system containing 41 μL ddH2O, 5 μL 10× CutSmart Buffer 2, 2 μL plasmid DNA, 1 μL EcoRI, and 1 μL XbaI. The reaction mixture was incubated at 37°C for 3 h, and the digested product was recovered by agarose gel

electrophoresis. Ligation was performed overnight at 16°C in a 20-μL system containing 1 μL linearized vector, 1 μL double-stranded DNA, 2 μL 10× buffer, 1 μL T4 DNA ligase, and 15 μL ddH2O. The ligation product was transformed into competent cells, and positive clones were confirmed by colony polymerase chain reaction and sequencing. High-purity plasmids were extracted using a plasmid extraction kit according to the manufacturer's protocol.

For lentiviral packaging, 293T cells in logarithmic growth phase were seeded in 10-cm dishes 24 h before transfection. When the cells reached 70%–80% confluence, the medium was replaced with serum-free medium. The target plasmid, pHelper 1.0, and pHelper 2.0 helper plasmids were mixed with transfection reagent (GENE, Shanghai, China) and added dropwise to the cells. After 6 h, the culture medium was replaced with complete medium, and the cells were cultured for an additional 48 h. Viral supernatants were collected, centrifuged at 4000 × g for 10 min at 4°C to remove debris, filtered through a 0.45-μm filter, concentrated by ultracentrifugation at 25000 rpm for 2 h, and resuspended in virus preservation medium. Viral titers were determined using gradient dilution infection of 293T cells and reverse transcription-polymerase chain reaction.

For HBVSMC transfection, cells were seeded 24 h before transfection and then incubated with lentiviral solution in serum-free medium containing polybrene (C0351; Beyotime, Shanghai, China). After overnight incubation, the medium was replaced with complete medium. Transfection efficiency was assessed by Western blotting. HBVSMCs were assigned to two groups: oe-Vector and oe-ACTN1. After transfection and incubation, cells

were collected for 5-ethynyl-2′-deoxyuridine proliferation assay, flow cytometric cell cycle analysis, wound-healing migration assay, F-actin staining, RhoA activation assay, and Western blotting.

Pharmacological inhibition with U0126 and Rhosin

To evaluate pathway involvement, ACTN1-overexpressing HBVSMCs were treated with the MEK inhibitor U0126-EtOH (S1102; Selleck, Shanghai, China) or the RhoA inhibitor Rhosin (S8988; Selleck, Shanghai, China). Cells were assigned to four groups: oe-Vector, oe-ACTN1, oe-ACTN1 + U0126, and oe-ACTN1 + Rhosin. For inhibitor treatment, oe-ACTN1 HBVSMCs were incubated with 10 μM U0126 or 20 μM Rhosin for 24 h. After treatment, cells were collected for 5-ethynyl-2′-deoxyuridine proliferation assay, flow cytometric cell cycle analysis, wound-healing migration assay, F-actin staining, RhoA activation assay, and Western blotting.

Culture and ACTN1 knockdown of iPSC-derived SMCs

HC-iPSC-SMCs and MMD-iPSC-SMCs were used to evaluate ACTN1 knockdown in a patient-relevant cell model. MMD-iPSC-SMCs were transfected with short hairpin RNA (shRNA) targeting ACTN1 or a non-targeting shRNA control. In this section, shRNA-ACTN1 denotes shRNA against ACTN1, and shRNA-NC denotes the non-targeting shRNA control.

Three independent shRNA-ACTN1 constructs were generated and evaluated for knockdown efficiency. The shRNA-ACTN1 sequences were as follows: shRNA-ACTN1 #1,

5′-CCGGCCTCAGGAGATCAATGGCAAACTCGAGTTTGCCATTGATCTCCTGAGGT TTTTG-3′; shRNA-ACTN1 #2, 5′-CCGGCGAAGAAATCGTGGATGGGAACTCGAGTTCCCATCCACGATTTCTTCGT TTTTG-3′; and shRNA-ACTN1 #3, 5′-CCGGCCAGACCTACCACGTCAATATCTCGAGATATTGACGTGGTAGGTCTGGT TTTTG-3′. The shRNA-NC sequence was 5′-CCGGCAACAAGATGAAGAGCACCAACTCGAGTTGGTGCTCTTCATCTTGTTGT TTTTG-3′.

For shRNA lentiviral vector construction, synthesized oligonucleotides containing the target sequence and restriction sites were annealed and ligated into a linearized vector digested with AgeI (ER1461; Takara, Japan) and EcoRI (1040A; Takara, Japan). Restriction digestion was performed in a 50-μL system containing 41 μL ddH2O, 5 μL 10× CutSmart Buffer 2, 2 μL plasmid DNA, 1 μL AgeI, and 1 μL EcoRI. Ligation was performed using T4 DNA ligase (2011A; Takara, Japan). Colony polymerase chain reaction and sequencing were used to identify positive clones.

For lentiviral packaging, 293T cells were transfected with the shRNA plasmid, pHelper 1.0, and pHelper 2.0 using transfection reagent (GENE, Shanghai, China). Viral supernatants were collected, filtered, concentrated, and resuspended in virus preservation medium. For cell transfection, MMD-iPSC-SMCs were incubated with the corresponding lentiviral solution. Puromycin (A1113803; Thermo Fisher Scientific, Pittsburgh, PA, USA) was used for selection where applicable.

Cells were assigned to four groups: HC-iPSC-SMC, MMD-iPSC-SMC, MMD-iPSC-SMC + shRNA-NC, and MMD-iPSC-SMC + shRNA-ACTN1. After transfection and incubation for 24 h, cells were collected for 5-ethynyl-2′-deoxyuridine proliferation assay, flow cytometric cell cycle analysis, Transwell migration assay, F-actin staining, RhoA activation assay, and Western blotting.

5-Ethynyl-2′-deoxyuridine proliferation assay

Cell proliferation was assessed using the BeyoClick EdU-594 Cell Proliferation Assay kit (C0078S; Beyotime, Shanghai, China) according to the manufacturer's instructions. Cells were seeded in 96-well plates at a density of 1×10^4 cells/well and labeled after the indicated treatments. A 2× 5-ethynyl-2′-deoxyuridine working solution was prepared by diluting the 10 mM stock solution with cell culture medium at a ratio of 1:500 to obtain a concentration of 20 μM. An equal volume of preheated 2× working solution was added to each well to reach a final concentration of 10 μM, followed by incubation at 37°C for 2 h.

After incubation, the culture medium was removed, and cells were fixed with 4% paraformaldehyde (P0099; Beyotime, Shanghai, China) at room temperature for 15 min. Cells were washed three times with PBS for 5 min each and permeabilized with PBS containing 0.3% Triton X-100 at room temperature for 15 min. After additional PBS washes, Click reaction solution was prepared and added according to the manufacturer's protocol. Nuclei were counterstained with Hoechst 33342 staining solution (C1025; Beyotime, Shanghai, China). Images were obtained using a fluorescence microscope or laser confocal

microscope (Zeiss LSM; Zeiss, Germany), and 5-ethynyl-2′-deoxyuridine-positive cells were quantified.

Flow cytometric cell cycle analysis

Cell cycle distribution was assessed using a cell cycle detection kit (KGA512; KeyGEN Biotech, Nanjing, China) according to the manufacturer's protocol. After the indicated treatments, cells were collected and prepared as a single-cell suspension. Cells were fixed in cold 70% ethanol overnight at 4°C. After fixation, cells were washed with PBS and stained with RNase/propidium iodide staining solution in the dark. The cell suspension was filtered through a nylon mesh before analysis. Cell cycle distribution was detected using a Beckman DxFlex flow cytometer (Beckman, USA), and the proportions of cells in the G0/G1, S, and G2/M phases were quantified.

Cell migration assay

For HBVSMC overexpression and pathway inhibition experiments, cell migration was assessed using a cell scratch assay. Cells were seeded and cultured until they reached a confluent monolayer. A linear scratch was made using a sterile pipette tip. Detached cells were removed by washing with PBS, and cells were cultured in the appropriate medium according to the experimental design. Images were obtained at 0 h and 24 h after scratching using a biological microscope (E200MV; Nikon Jiangnan Optical Instrument, Nanjing, China). Relative migration was quantified by measuring wound closure.

For iPSC-SMC knockdown experiments, cell migration was assessed using a transwell assay. Cells were digested, centrifuged, and resuspended in serum-free medium at a density of 2.5 × 10^5 cells/mL. A 100-µL cell suspension was added to the upper chamber, and 600 µL of medium containing FBS was added to the lower chamber. After incubation at 37°C for 24 h, non-migrated cells were removed. Migrated cells were fixed with 4% paraformaldehyde and stained with 0.1% crystal violet staining solution (KGA229; KeyGEN Biotech, Nanjing, China). The inserts were washed and air-dried, and migrated cells were imaged under an inverted microscope.

F-actin staining

F-actin organization was assessed using Actin-Tracker Green-488 (C2201S; Beyotime, Shanghai, China). After the indicated treatments, cells were fixed with 4% paraformaldehyde, washed with PBS, and permeabilized. Cells were then incubated with Actin-Tracker Green-488 according to the manufacturer's instructions. Nuclei were counterstained with 4′,6-diamidino-2-phenylindole (DAPI; C0060; Solarbio, Beijing, China). Fluorescence images were obtained using a fluorescence microscope or laser confocal microscope (Zeiss LSM; Zeiss, Germany). F-actin organization and stress fiber formation were assessed from representative fields.

RhoA activation assay

RhoA activity was assessed using a RhoA Activation Assay Kit (80601; NewEast Biosciences, PA, USA) according to the manufacturer's instructions. After the indicated

treatments, cells were lysed, and active guanosine triphosphate-bound RhoA (RhoA-GTP) was isolated using the pull-down reagent supplied with the kit. RhoA-GTP and total RhoA (T-RhoA) were detected by Western blotting. RhoA activation was expressed as the ratio of RhoA-GTP to T-RhoA.

Western blotting

After treatment, cells were lysed using RIPA lysis buffer (KGP702-100; KeyGEN BioTECH, Nanjing, China) supplemented with PMSF (97064-898; Amresco, VWR International, OH, USA). Protein concentration was determined using a BCA protein assay kit (KGP902; KeyGEN BioTECH, Nanjing, China). Equal amounts of protein were mixed with loading buffer (WB2001; NCM Biotech, Suzhou, China), separated by SDS-PAGE using 30% acrylamide (ST003; Beyotime, Shanghai, China), ammonium persulfate substitute (ST005; Beyotime), 1.0 mol/L Tris pH 6.8 (ST768; Beyotime), 1.5 mol/L Tris pH 8.8 (ST789; Beyotime), 10% SDS (BL517A; Biosharp, Hefei, China), and TEMED (17919; Thermo Fisher Scientific), and transferred to PVDF membranes (0.2 µm; ISEQ00010; Millipore, Schwalbach, Germany) using a Western blotting system (Criterion electrophoresis cell and Trans-Blot transfer cell; Bio-Rad). StarSignal Western Protein Marker (10–200 kDa; M227-01; GenStar, Beijing, China) was used as the protein marker.

After blocking, membranes were incubated with primary antibodies against ACTN1 (ab68194; Abcam), RhoA (ab187027; Abcam), ROCK (ab134181; Abcam), MYPT1 (2634; Cell Signaling Technology), p-MYPT1 (T696; 5163; Cell Signaling Technology), MLC2 (8505; Cell Signaling Technology), p-MLC2 (Ser19; 3671; Cell Signaling Technology),

LIMK1 (ab119084; Abcam), p-LIMK1 (T508; ab194798; Abcam), cofilin (5175; Cell Signaling Technology), p-cofilin (Ser3; 3311; Cell Signaling Technology), MEK1/2 (9122; Cell Signaling Technology), p-MEK1/2 (Ser217/221; 9121; Cell Signaling Technology), ERK1/2 (9102; Cell Signaling Technology), p-ERK1/2 (Tyr202/Tyr204; 9101; Cell Signaling Technology), and GAPDH (ab8245; Abcam).

After washing with TBST prepared from TBST powder (G0001; Servicebio, Wuhan, China), membranes were incubated with goat anti-rabbit IgG H&L (HRP; ab205718; Abcam) or goat anti-mouse IgG H&L (HRP; ab205719; Abcam). Protein bands were visualized using enhanced chemiluminescence reagent (32209; Thermo Fisher Scientific) and imaged using a chemiluminescence imaging system (Tanon 5200; Tanon, Shanghai, China). GAPDH was used as the loading control. For phosphorylation analyses, phosphorylated protein levels were normalized to the corresponding total protein where applicable.

Statistical analysis

Data are presented as mean ± standard deviation. Comparisons between two groups were performed using Student's t-test. Comparisons among more than two groups were performed using one-way analysis of variance followed by Tukey's post hoc test. A two-sided P value < 0.05 was considered statistically significant.

Table S1. Clinical characteristics and sample information of the patients with MMD.

Characteristic	Hemorrhagic (n=20)<br>Mean ± SD1	Ischemic (n=20)<br>Mean ± SD1	P-value2
**Sex (M:F)**			0.752
F	10 (50%)	11 (55%)	
M	10 (50%)	9 (45%)	
**Age, y**	34 ± 10	33 ± 7	0.739
**Hypertension**			0.376
NO	16 (80%)	18 (90%)	
YES	4 (20%)	2 (10%)	
**Diabetes mellitus**			
NO	20 (100%)	20 (100%)	
**Coronary heart disease**			
NO	20 (100%)	20 (100%)	
**Dyslipidemia**			
NO	20 (100%)	20 (100%)	
**Duration (Months)**	14 ± 27	24 ± 36	0.348
**Suzuki Stage**			
**Left**			0.817
No findings	1 (5.0%)	1 (5.0%)	
1	2 (10%)	2 (10%)	
2	5 (25%)	8 (40%)	
3	8 (40%)	4 (20%)	
4	2 (10%)	2 (10%)	
5	2 (10%)	3 (15%)	
6	0 (0%)	0 (0%)	
**Right**			0.272
No findings	1 (5.0%)	0 (0%)	
1	4 (20%)	0 (0%)	
2	5 (25%)	6 (30%)	
3	6 (30%)	11 (55%)	
4	2 (10%)	2 (10%)	
5	1 (5.0%)	1 (5.0%)	
6	1 (5.0%)	0 (0%)	

1n (%); Mean ± SD

2Pearson's Chi-squared test; Welch Two Sample t-test; NA

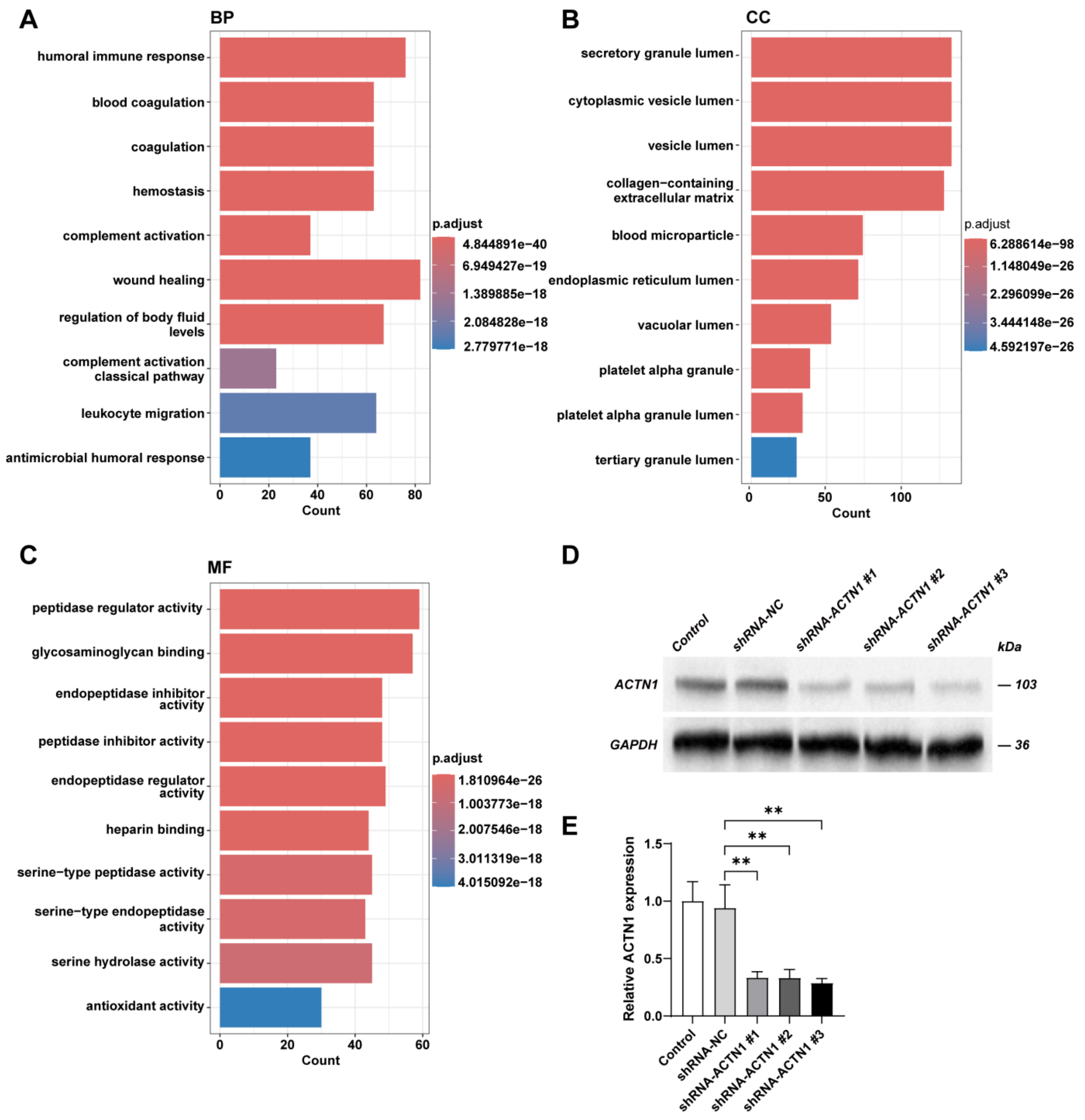

Figure S1: Functional enrichment analysis of differentially expressed proteins and validation of ACTN1 knockdown efficiency.

A–C. Gene Ontology (GO) enrichment analysis of the differentially expressed proteins between patients with moyamoya disease (MMD) and healthy controls (HCs). **A**. Top enriched biological process (BP) terms. **B**. Top enriched cellular component (CC) terms. **C**. Top enriched molecular function (MF) terms. Bar length represents protein count, and color indicates adjusted *P* value. **D**. Western blot analysis of ACTN1 expression in control cells,

shRNA-NC cells and cells transfected with three independent shRNAs targeting ACTN1. GAPDH was used as the loading control. **E**. Quantification of relative ACTN1 expression in D. Among the three shRNAs tested, shRNA-ACTN1 #3 showed the strongest knockdown efficiency and was selected for subsequent experiments. Data are mean ± SD from three independent experiments. $^{**}P < 0.01$.

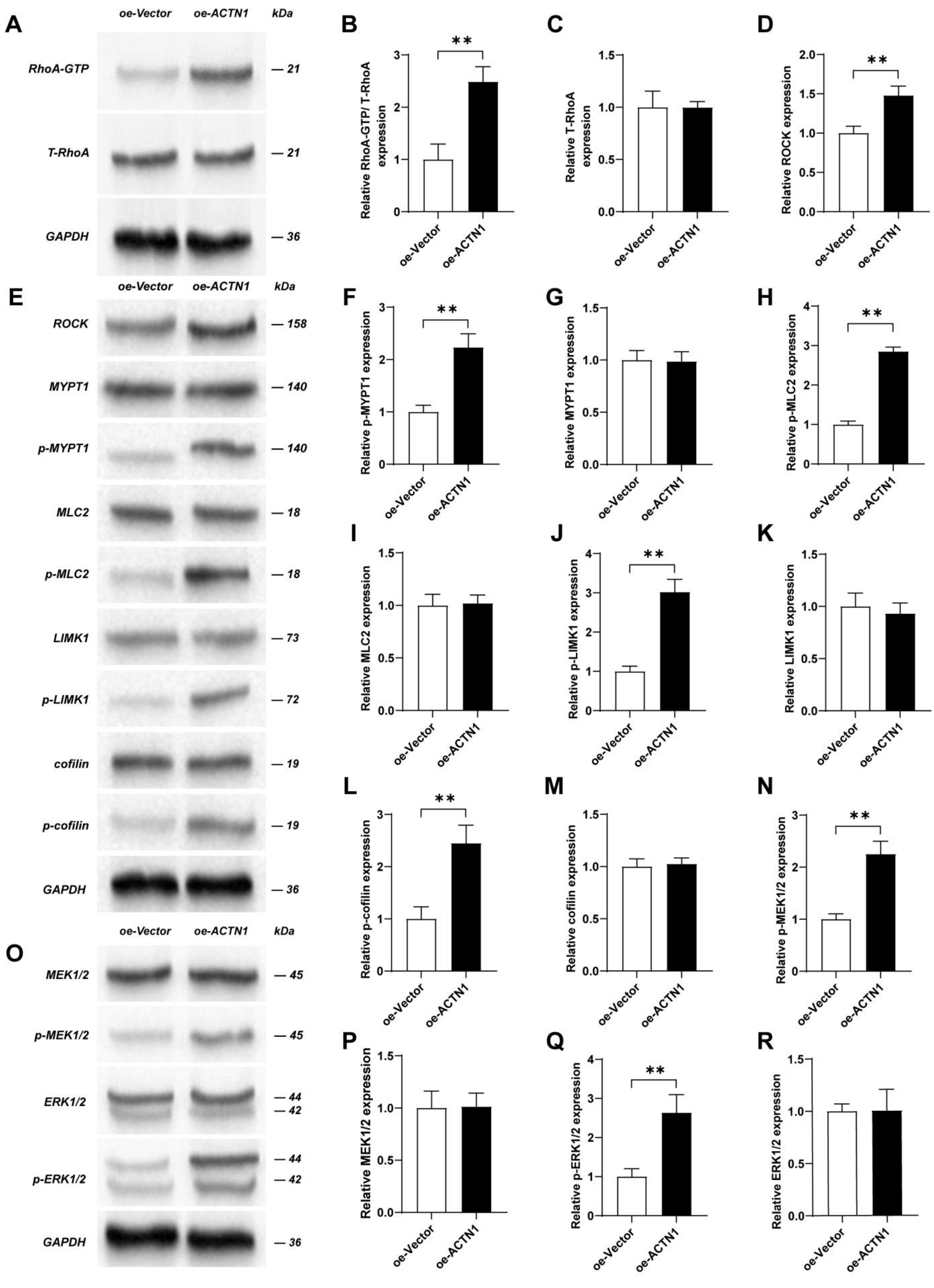

Figure S2: ACTN1 overexpression activates RhoA/ROCK and MEK/ERK signaling in HBVSMCs.

A.Western blots of RhoA-GTP, total RhoA (T-RhoA) and GAPDH in oe-Vector and oe-ACTN1 HBVSMCs. **B–D**. Quantification of RhoA-GTP/T-RhoA, T-RhoA and ROCK. **E**. Western blots of ROCK, MYPT1, p-MYPT1, MLC2, p-MLC2, LIMK1, p-LIMK1, cofilin, p-cofilin and GAPDH. **F–M**. Quantification of p-MYPT1, MYPT1, p-MLC2, MLC2, p-LIMK1, LIMK1, p-cofilin and cofilin. **N**. Quantification of p-MEK1/2. **O**. Western blots of MEK1/2, p-MEK1/2, ERK1/2, p-ERK1/2 and GAPDH. **P–R**. Quantification of MEK1/2, p-ERK1/2 and ERK1/2. Data are mean ± SD from three independent experiments. $^{**}P < 0.01$.

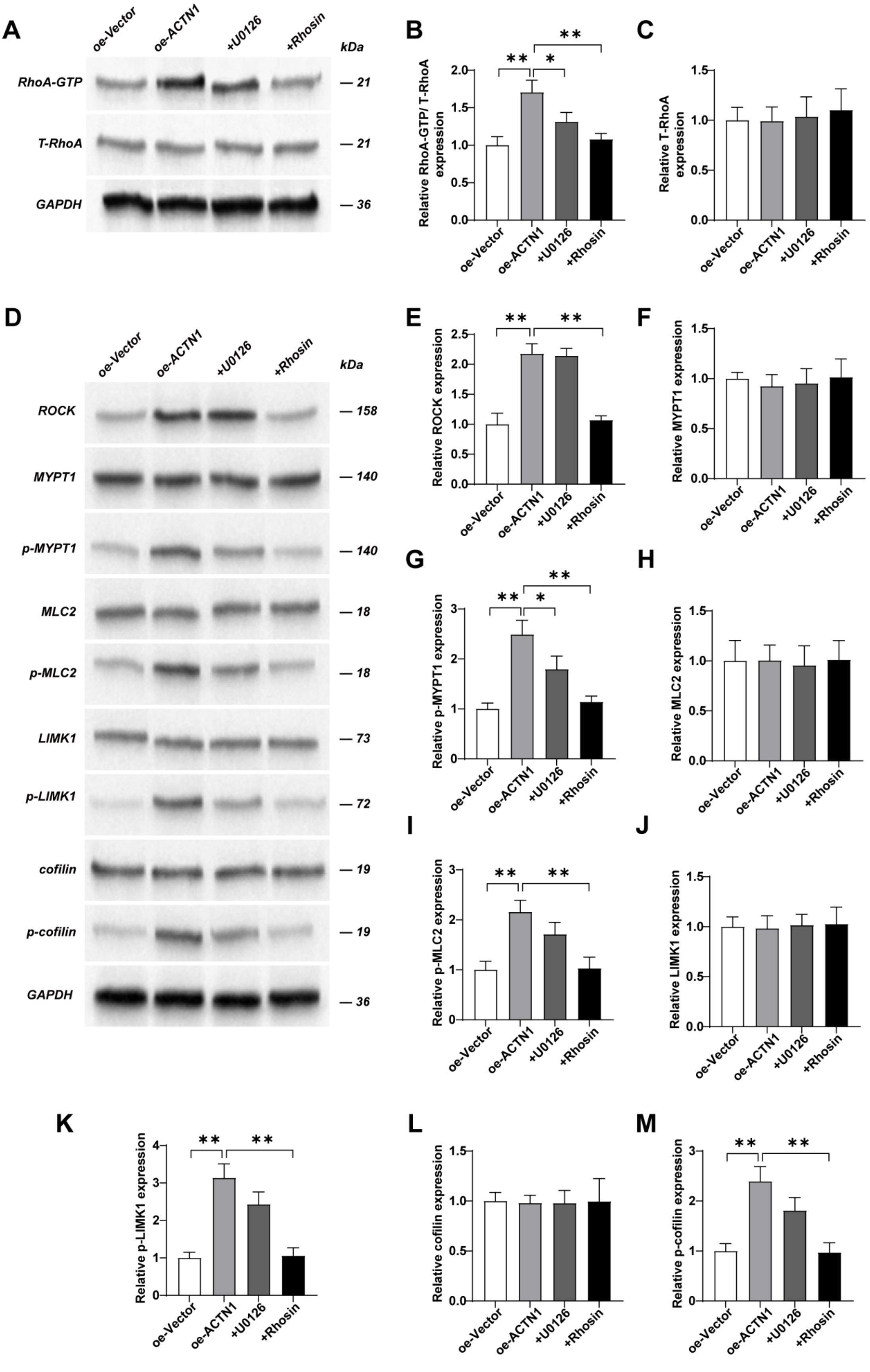
A
oe-Vector
oe-ACTN1
+U0126
+Rhosin
kDa
RhoA-GTP
T-RhoA
GAPDH
21
21
36
B
Relative RhoA-GTP/ T-RhoA expression
C
Relative T-RhoA expression
D
ROCK
MYPT1
p-MYPT1
MLC2
p-MLC2
LIMK1
p-LIMK1
cofilin
p-cofilin
GAPDH
158
140
140
18
18
73
72
19
19
36
E
Relative ROCK expression
F
Relative MYPT1 expression
G
Relative p-MYPT1 expression
H
Relative MLC2 expression
I
Relative p-MLC2 expression
J
Relative LIMK1 expression
K
Relative p-LIMK1 expression
L
Relative cofilin expression
M
Relative p-cofilin expression
**
*

Figure S3: Effects of RhoA and MEK inhibition on RhoA/ROCK-associated signaling in ACTN1-overexpressing HBVSMCs.

+U0126 and +Rhosin denote oe-ACTN1 HBVSMCs treated with the MEK inhibitor U0126 or the RhoA inhibitor Rhosin, respectively; oe-Vector and oe-ACTN1 HBVSMCs were used as controls. **A**. Western blots of RhoA-GTP, T-RhoA and GAPDH. **B**, **C**. Quantification of RhoA-GTP/T-RhoA and T-RhoA. **D**. Western blots of ROCK, MYPT1, p-MYPT1, MLC2, p-MLC2, LIMK1, p-LIMK1, cofilin, p-cofilin and GAPDH. **E–M**. Quantification of ROCK, MYPT1, p-MYPT1, MLC2, p-MLC2, LIMK1, p-LIMK1, cofilin and p-cofilin. Data are mean ± SD from three independent experiments. $^{*}P < 0.05$, $^{**}P < 0.01$.

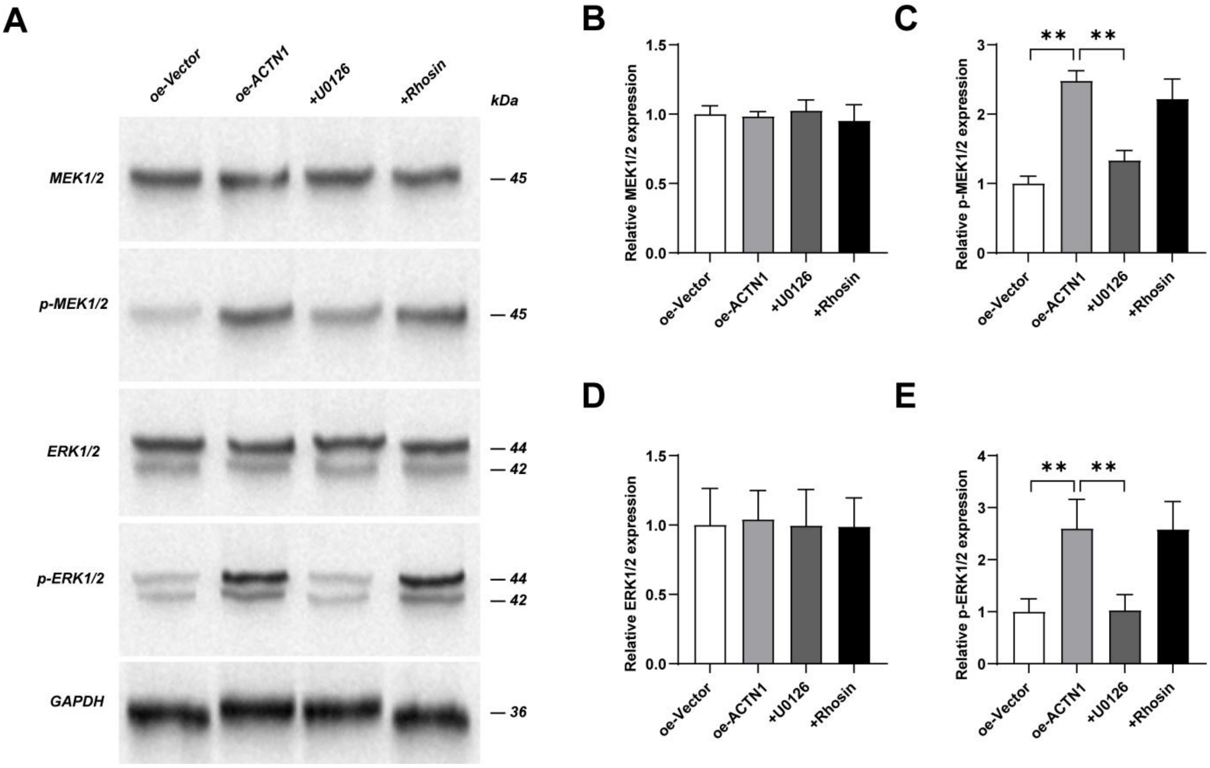

Figure S4: Effects of RhoA and MEK inhibition on MEK/ERK signaling in ACTN1-overexpressing HBVSMCs.

+U0126 and +Rhosin denote oe-ACTN1 HBVSMCs treated with the MEK inhibitor U0126 or the RhoA inhibitor Rhosin, respectively; oe-Vector and oe-ACTN1 HBVSMCs were used

as controls. **A**. Western blots of MEK1/2, p-MEK1/2, ERK1/2, p-ERK1/2 and GAPDH. **B–E**. Quantification of MEK1/2, p-MEK1/2, ERK1/2 and p-ERK1/2. Data are mean ± SD from three independent experiments. $^{**}P < 0.01$.

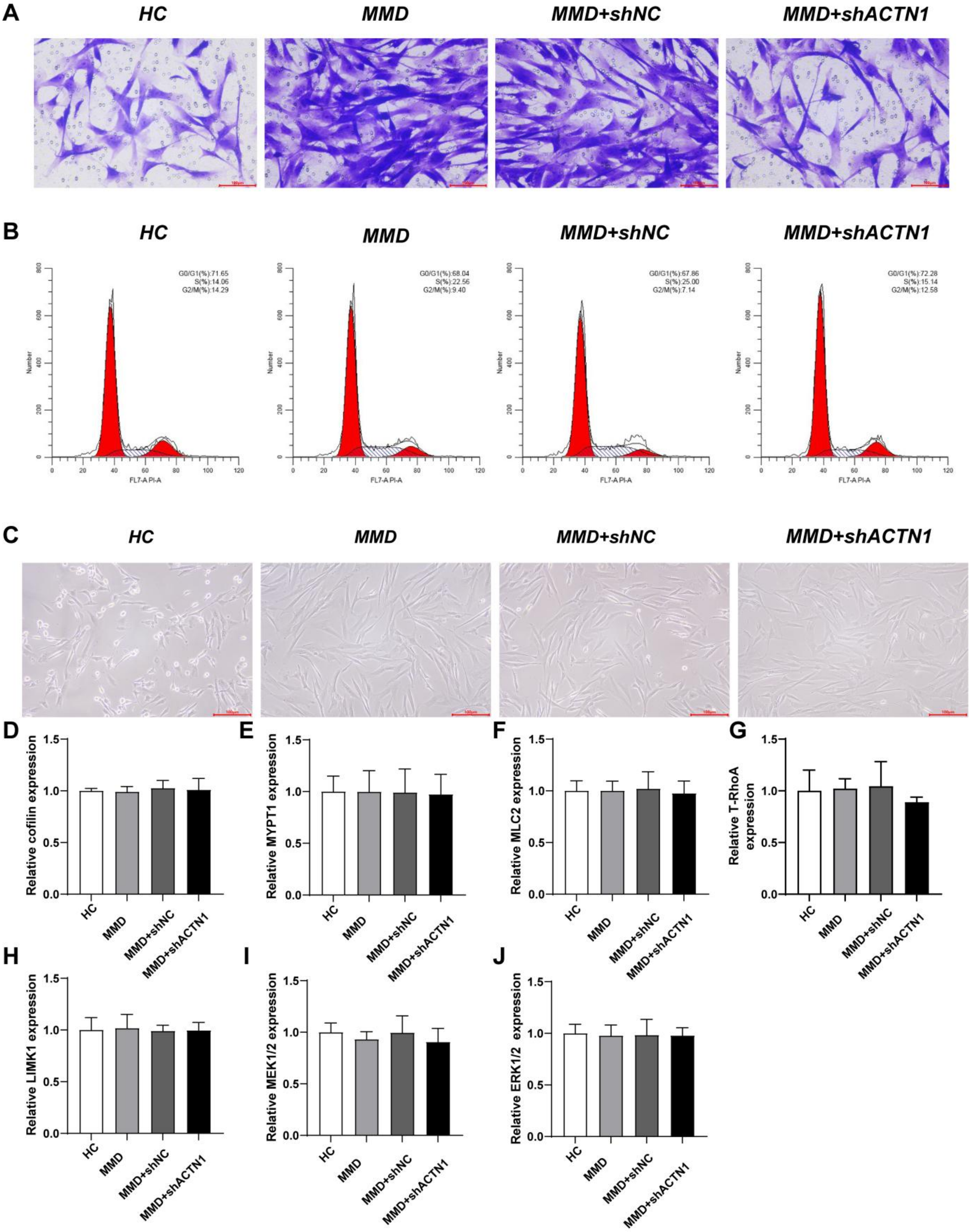

Figure S5: Additional phenotypic images and total protein controls in patient-derived MMD-iPSC-SMCs after ACTN1 knockdown.

HC and MMD denote HC-iPSC-SMCs and MMD-iPSC-SMCs, respectively. MMD+shNC denotes MMD-iPSC-SMCs transfected with a non-targeting shRNA, and MMD+shACTN1 denotes MMD-iPSC-SMCs transfected with shRNA against ACTN1. **A**. Transwell migration images. Scale bar = 100 μm. **B**. Flow cytometric cell-cycle analysis. **C**. Bright-field images showing morphological changes. Scale bar = 100 μm. **D–J**. Quantification of cofilin, MYPT1, MLC2, T-RhoA, LIMK1, MEK1/2 and ERK1/2 expression. Data are mean ± SD from three independent experiments.
